# ﻿Biological resources of Eunotidae, Herbertiidae, Pteromalidae and Eulophidae (Hymenoptera, Chalcidoidea) from the Altun Mountain National Nature Reserve, China, with description of ten new species

**DOI:** 10.3897/zookeys.1233.140823

**Published:** 2025-03-26

**Authors:** Ning Kang, Hong-Ying Hu

**Affiliations:** 1 Xinjiang Key Laboratory for Ecological Adaptation and Evolution of Extreme Environment Biology, College of Life Sciences, Xinjiang Agricultural University, Urumchi, China; 2 College of Life Science and Technology, Xinjiang University, Urumqi, Xinjiang 830046, China; 3 Xinjiang Key Laboratory of Biological Resources and Genetic Engineering, Xinjiang University, Urumqi, Xinjiang 830046, China

**Keywords:** Alpine, biological control, checklist, Eulophidae, Eunotidae, Herbertiidae, new species, population density, Pteromalidae, species abundance

## Abstract

In this study, we explore the biological resources of Eunotidae, Herbertiidae, Pteromalidae and Eulophidae in the Altun Mountain Nature Reserve, Xinjiang, China. Sixty-one species are listed and we described ten new species, including *Eunotuscaeruleus* Kang & Hu, **sp. nov.** and *Eunotusargenteus* Kang & Hu, **sp. nov.** of Eunotidae, *Herbertiaaltunensis* Kang & Hu, **sp. nov.** of Herbertiidae, *Thinodytessplendens* Kang & Hu, **sp. nov.**, *Erdoesinamaculata* Kang & Hu, **sp. nov.**, *Homoporusflavus* Kang & Hu, **sp. nov.** and *Stenomalinaviridis* Kang & Hu, **sp. nov.** of Pteromalidae and two new species *Diaulinopsisaltunensis* Kang & Hu, **sp. nov.** and *Hyssopusaltunensis* Kang & Hu, **sp. nov.** of Eulophidae. Detailed illustrations of all new species are included to support identification and further study.

## ﻿Introduction

Pteromalidae and Eulophidae are among the largest families within the superfamily Chalcidoidea (Insecta, Hymenoptera), notable for their vast diversity and global distribution. Currently, over 4000 species across 33 subfamilies and 640 genera of Pteromalidae have been documented worldwide, while Eulophidae encompasses more than 6000 species belonging to 328 genera across 5 subfamilies. In China alone, 388 species of 51 genera in Eulophidae, 110 genera and over 450 species in Pteromalidae have been recorded (Noyes 2019). Through years of research on morphological combining molecular phylogeny, the taxonomic status of the families Pteromalidae and Eulophidae has been changed (Burks et al. 2022; Cruaud et al. 2022). The two subfamilies Eunotinae Ashmead, 1904 and Herbertiinae Bouček, 1988 of Pteromalidae have been elevated to the family level of Eunotidae and Herbertiidae accordingly. At present, 23 genera and 17 species have been recorded in Eunotidae, of which 8 species have been recorded in China, and 3 genera and 10 species of Herbertiidae have been recorded worldwide, with only one species recorded in China (Bouček 1988). These families primarily consist of species which parasitize primarily hosts in the orders Lepidoptera, Coleoptera, Diptera, Hemiptera, as well as other arthropods (Cruaud et al. 2022). Their role in the biological control of agricultural pests highlights their ecological importance and biogeographical relevance.

Although Pteromalidae, Eunotidae, Herbertiidae and Eulophidae are widely distributed and commonly found in diverse habitats, they tend to show reduced species diversity and larger body sizes in alpine environments (Kang et al. 2023). To date, there are few records of these families from such regions (Table 1), with most collections concentrated in the Himalaya Mountains.

**Table 1.** Records of high altitude Pteromalidae and Eulophidae.

Family	Group	Alpine area	Reference
Pteromalidae	*Tridymus* sp.	Canadian Arctic	(Kevan 1973)
	*Asaphessuspensus*, *Dibrachyscavus*, *Pachyneuronnelson*, *Schizonotuslatus* et al.	Ladakh region of the Trans-Himalaya	(Sureshan 2012)
	*Herbertiaindica*, *Pteromaluspuparum*	Kashmir Himalaya	(Bhat et al. 2017)
	*Asaphessuspensus*, *Ammeiapulchella*, *Halticopterinatriannulata* et al.	Karatau Ridge and adjacent area in Western Tien Shan	(Dzhanokmen 2017)
	*Sphaeripalpus*, *Halticoptera*, *Lamprotatus*, *Thektogaster*	Tibet Plateau	(Xiao and Huang 2004)
Eulophidae	*Chrysocharis*	Italian Alps, high altitude of India	(Khan 1984; Hannes and Hansson 1997)
	*Diglyphusisaea*	Himalaya Mountains,	(Sha et al. 2007)
	*Neotrichoporoide*, *Tamarixia*, *Pronotalia*	Georgia	(Japoshvili and Kostjukov 2016)
	*Diglyphus*, *Cirrospilus*, *Hemiptarsenus*	North-western Himalayas, India	(Kumar and Sharma 2016)

The Altun Mountain National Nature Reserve, situated in the southeastern Xinjiang Autonomous Region of northwest China, lies at the northern edge of the Tibetan Plateau and the southern boundary of the Tarim Basin. This reserve is distinguished by its unique extreme environmental conditions, including low temperatures, extremely arid, strong winds and high ultraviolet radiation. It features a variety of habitats, such as expansive sandy and gravel deserts, wetlands, and alpine steppes, with an average elevation exceeding 4500 m. These harsh conditions promote the development of unique species.

In this study, we enhance our understanding of Pteromalidae, Eunotidae, Herbertiidae and Eulophidae within the Altun Mountain Nature Reserve by listing 61 species, including 10 ten new species described and illustrated. This work marks a crucial advancement in addressing the taxonomic and ecological complexities of these families in an alpine region that has been historically overlooked in entomological studies.

## ﻿Material and methods

**Figure 1.**
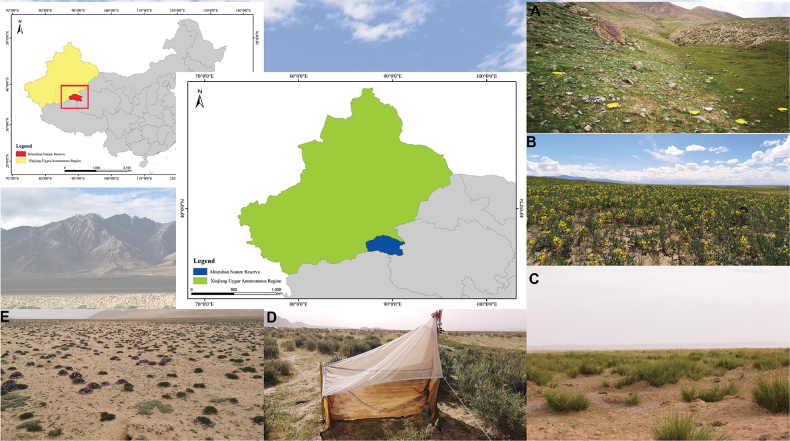
**A–F** Location and habitat of protected area sampling sites **A** geographical location of the Altun Mountain National Nature Reserve **B** alpine meadow, 4240 m **C** plateau desert, 4050 m (main vegetation is *Thermopsisalpina* (Pall.) Ledeb.) **D** plateau wetland, 3890 m (main vegetation is *Neotriniasplendens* (Trin.) M. Nobis, P. D. Gudkova & A. Nowak) **E** alpine wetland, 3450 m (main vegetation is *Myricaria* sp.) **F** gravel desert, 3790 m (main vegetation is *Oxytropis* sp.).

All the examined specimens were collected by net sweeping, yellow pan traps, and Malaise traps in July from 2019 to 2021; yellow pans were left from 2 to 24 h at each site, and alcohol in Malaise traps was changed every 10 (±5) days to 1 month. The specimens were sorted and preserved in absolute ethanol immediately, stored at -20 °C in the lab. Selected specimens of both sexes were first air dried, then critical point dried (CPD, K850, Quorum), point-mounted or slide-mounted with labels, and then examined under a Nikon SMZ745T stereomicroscope using the available keys (Bouček 1988; Trjapitzin, 1989; Bouček and Rasplus 1991; Huang and Xiao 2005). Habitus photographs were taken with a Nikon D7000 digital camera connected to a Nikon SMZ25 stereomicroscope, at least 35 images were used for stacking to achieve high-quality images, and plates were compiled using Adobe Illustrator software. All specimens were deposited in the Insect Collection of the College of Life Science and Technology, Xinjiang University, Urumqi, Xinjiang, China (ICXU) (Fig. 1).

The taxonomic terminology and abbreviations follow relevant professional monographs (Graham 1969; Bouček 1988; Bouček and Rasplus 1991; Gibson et al. 1997; Huang and Xiao 2005). The following abbreviations are used in the text:

**F1–6** Funicle segment from the first to the sixth;

**POL** Distance between the posterior ocelli;

**OOL** Distance between the eye margin and the adjacent posterior ocellus;

**OCL** Distance between the posterior ocellus and the occipital margin;

**Gt_1-7_** Gastral tergite segment from the first to the seventh.

## ﻿Results

### ﻿Eunotidae

#### ﻿ Eunotus caeruleus

Taxon classificationAnimaliaHymenoptera﻿Eunotidae

Kang & Hu sp. nov.

1F950E85-4EC7-5BB9-9378-7EA33755A010

https://zoobank.org/75ACC7E5-C7F5-49A0-87EE-24A960A866E5

Fig. 2A–F

##### Type material.

***Holotype***. • ♀, point-mounted, China, Xinjiang, Ruoqiang County, Altun Mountain Nature Reserve, 38°4'22.5288"N, 89°7'10.7472"E, Altitude: 3681.55 m, 13.VII.2020, Coll. Ning Kang by sweeping net. ***Paratypes*.** • 1 ♀, 1 ♂, card mounted, same data as holotype except 15.VII.2020; • 3 ♀♀, 5 ♂♂, card mounted, 16.VII.2021. Coll. Shun-Gang Luo, Ning Kang, Hong-Ying Hu (All deposited in ICXU).

##### Description.

**Figure 2.**
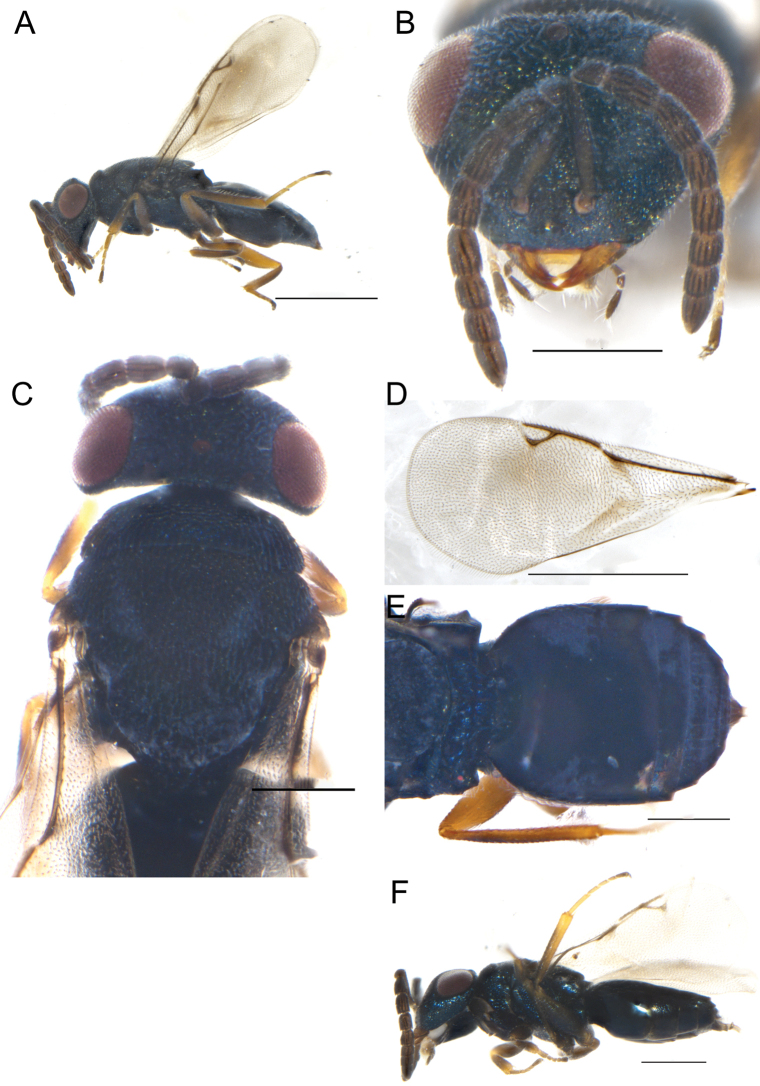
**A–F***Eunotuscaeruleus* Kang & Hu, sp. nov., female holotype **A** habitus, lateral view **B** head, frontal view **C** mesoscutum, dorsal view **D** forewing E gaster, dorsal view **F** Male, body, lateral view. Scale bars: 500 μm (**A, D**); 200 μm (**B, C, E, F**).

Female. Length 1.3 mm. Body dark blue (Fig. 2A), eyes dark red, antenna dark brown. Legs with all coxae same color as the body; femora and tibiae dark brown, except trochanter and their apices deep yellow, forewing transparent covered densely with setae (Fig. 2D).

Head, distinct inverted triangle, in frontal view 1.45× as broad as high (Fig. 2B), the inner margin of the compound eye curved outward, eye height 0.61× as long as interocular distance, 0.85× as long as scape. Clypeus margin straight, without tooth, mandible bidentate, dark yellow. Antenna located before the lower eye margin and the distance from median ocellus by 3.96× height of the distance from clypeus margin, scape not reaching anterior ocellus, pedicel short and square, 0.6× as long as F1; F1-F4 all longer than width, funicle length 1.5× as long as width, each funicle segment with two rounds of dark plate-shaped sensilla; clava shorter than the combined length of the last two funicle segments, 3.35× as long as broad; length of flagellum and pedicel combined longer than head width (0.9×). Head in dorsal view 3.18× as broad as long, POL 4.2× OOL.

Mesosoma not distinctly convex, covered with reticulated and dense engraving, notauli distinct and complete (Fig. 2C). Pronotum 0.7× as long as mesoscutum length, anteriorly not margined; scutellum 0.6× as long as mesoscutum, frenal line absent. Propodeum 0.35× as long as scutellum, reticulation irregular, the rear of both sides protruded, nucha short (Fig. 2E). Forewing 2.2× as long as broad, covered with dense setae, without speculum and marginal fringe, marginal vein 1.04× as long as postmarginal vein, postmarginal vein 1.15× as long as stigmal vein, the angle between stigmal vein and postmarginal vein 40° (Fig. 2D).

Gaster 1.6× as long as broad, shorter than the combined length of head and mesosoma, Gt_1_ distinctly longer than other tergites, 0.56× as long as the gaster, smooth without distinct markings (Fig. 2E).

**Male.** Length 1.0 ± 0.2 mm, *N* = 5 (Fig. 2F), similar to female in body color and habitus, but differs as follows. The last two flagella significantly shorter than the first two funiculus, square and short. Gaster short and flat.

##### Host.

Unknown.

##### Etymology.

“*caeruleus*” means dark blue, signifying the dark blue body color of the female species.

##### Diagnosis.

The new species is morphologically similar to *E.parvulus*, but distinctly different in several key traits: the body color of the latter is dark green and gaster dark brown, while the color of new species is dark blue; POL: OOL of the latter is 4.5 while the new species is 4.2; pedicel 2× as long as width, longer than F1, and significantly longer than the new species; all funicle segments broader than long and transverse while for the new species they are obviously longer than width; clava 2× as long as broad, obviously shorter than the new species; propodeum with complete median carina and costulae, while the new species is covered with irregular reticulation; and Gt_1_ 0.86× as long as gaster, significantly longer than the Gt_1_ of the new species.

#### ﻿ Eunotus argenteus

Taxon classificationAnimaliaHymenopteraEunotidae

Kang & Hu sp. nov.

DC55516F-762E-5572-A533-4259DE17E6BD

https://zoobank.org/3F61ECBE-C822-46A3-8978-67B0EEE8002F

Fig. 3A–G

##### Type material.

***Holotype*.** • ♀, card mounted, Xinjiang, Ruoqiang, Altun Mountain Nature Reserve, 36°58'10.8984"N, 90°14'44.916"E, Altitude: 4021.95 m, 16.VII.2021. Coll. Ning Kang. ***Paratypes*.** • ♀, 38°0'1.9512"N, 89°0'31.3164"E, Altitude: 3717.15 m, 19.VII.2020. Coll. Ning Kang, by sweeping net; • 2 ♀♀, 38°0'17.5428"N, 88°53'20.5044"E, Altitude: 3771.58 m, 19.VII.2021. Coll. Ning Kang, by sweeping net (All deposited in ICXU).

##### Description.

**Figure 3.**
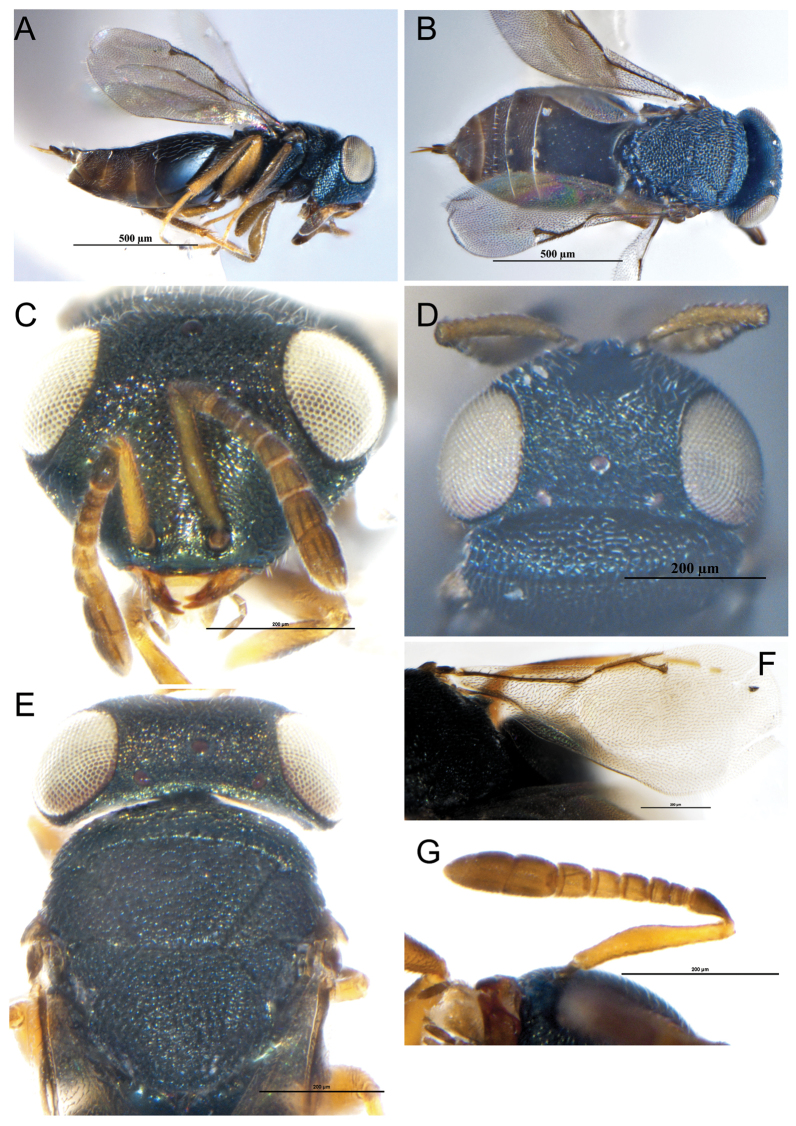
**A–G***Eunotusargenteus* Kang & Hu, sp. nov., female holotype **A** habitus, lateral view **B** habitus, dorsal view **C** head, frontal view **D** head, dorsal view **E** mesoscutum, dorsal view **F** forewing **G** antenna. Scale bars: 500 μm (**A, B**); 200 μm (**C−G**).

**Female.** Length 1.3 mm (Fig. 3A), body black green, gaster dark brown; ocelli silver white, eyes deep red; antenna light brown, pedicel and clava dark brown; legs brown, coxae same color with body, tarsi yellow, distal tibiae dark brown; forewing hyaline, venation dark brown.

Head 1.45× as broad as high (Fig. 3C), the inner margin of eyes curved laterally, the distance between eyes 1.43× as their height. Antennal insertion below the lower ocular line, distance from upper margin of torulus to lower margin of anterior ocellus 7.2× distance from lower margin of torulus to lower margin of clypeus; scape 1.12× as long as eye height, not reaching anterior ocellus, pedicel longer than the combined length of first two funicular segments; five funicular segments(Fig. 3G), the first two segments transverse, the last three segments gradually lengthened, clava clavate, length of flagellum and pedicel combined longer than head width (0.77×). Head in dorsal view, 2.75× as wide as long, POL 3.9× OOL (Fig. 3D); Head in lateral view, 0.66× as broad as height, malar sulcus not distinct, eye height 1.24× malar space.

Head 1.25× as broad as mesosoma. Mesosoma not distinctly convex, pronotum 0.35× as long as mesoscutum (Fig. 3B), mesoscutum 0.85× as long as scutellum, notauli complete and distinctly straight, scutellum 1.74× as broad as long, frenal line absent (Fig. 3E). Fore wing 2.25× as long as broad (Fig. 3F), covered with dense hair, marginal vein 1.48× postmarginal vein, postmarginal vein 0.94× stigmal vein, stigmal vein slightly capitate.

Gaster sessile, abdomen flattened, 1.35× as long as thorax, approximately as long as broad. Gt_1_ 0.5× as long as the gaster.

**Male.** Unknown.

##### Hosts.

Unknown.

##### Etymology.

‘*argenteus*’ means silver, as in the color of the compound eyes of this species.

### ﻿Herbertiidae

#### ﻿ Herbertia altunensis

Taxon classificationAnimaliaHymenoptera﻿Herbertiidae

Kang & Hu sp. nov.

735570EA-76A2-54CB-9BB4-959701F2DE43

https://zoobank.org/2B750C67-7087-4C99-BED6-9B4DCC4EAA44

Fig. 4A–G

##### Material examined.

***Holotype*.** • ♀, China: Xinjiang, Ruoqiang, Altun Mountain Nature Reserve, 38°4'22.5288"N, 89°7'10.7472"E, Altitude: 3681.55 m, 13.VII.2020. Coll. Ning Kang, by sweeping net; ***Paratypes*.** • 2 ♀♀, 13.VII.2020. same locality, Coll. Ning Kang; • 1 ♀, 19.VII.2021. same locality, Coll. Ning Kang (All deposited in ICXU).

##### Description.

**Figure 4.**
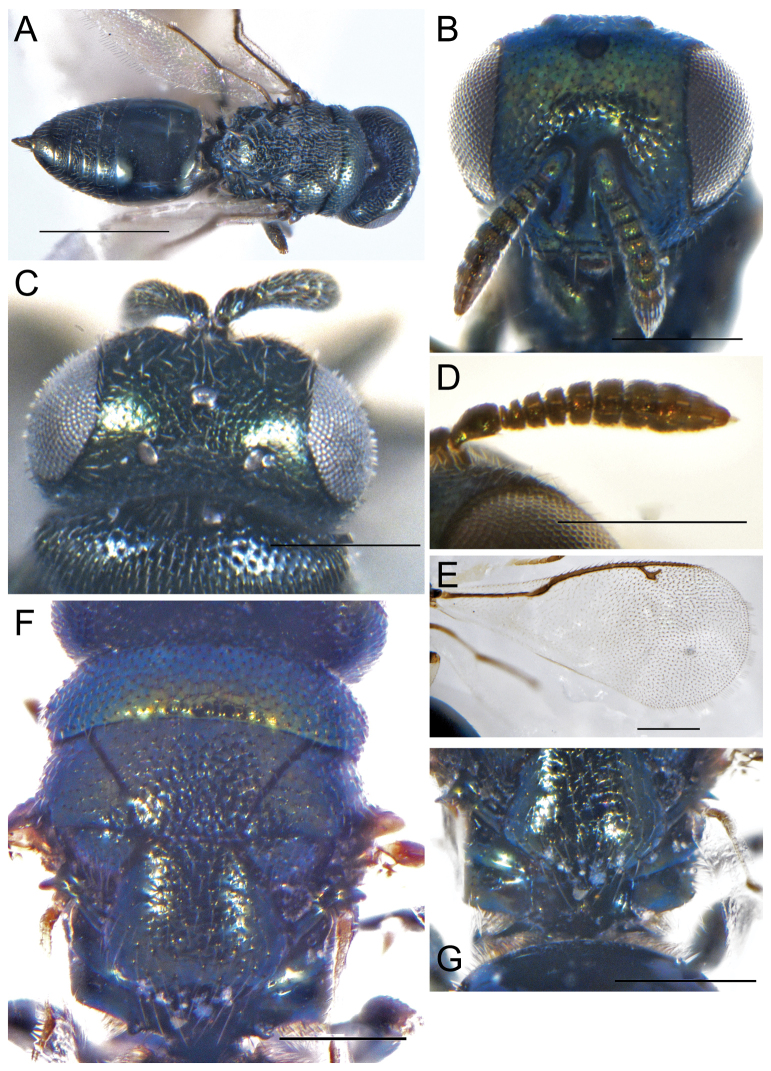
**A–G***Herbertiaaltunensis* Kang & Hu, sp. nov., female holotype **A** habitus, dorsal view **B** head, frontal view **C** head, dorsal view **D** antenna **E** forewing **F** mesosoma, dorsal view **G** propodeum, dorsal view. Scale bars: 500 μm (**A**); 200 μm (**B−D, F**); 250 μm (**E, G**).

**Female.** Length 1.5 mm (Fig. 4A); body dark aeneous, with green metallic reflection, antenna concolorous with body, clava dark brown, eyes and ocelli dark red, coxae and femurs concolorous with body, tibiae light brown, forewing hyaline, venation dark brown.

Head in frontal view 1.25× as wide as high (Fig. 4B), eyes separated by 1.24× their height. Antennal insertion below lower ocular line, distance from upper margin of torulus to lower margin of anterior ocellus 5.1× distance from lower margin of torulus to lower margin of clypeus, clypeus margin slightly concave. Antenna stubby (Fig. 4D), length of flagellum and pedicel combined longer than head width (0.68×), each funicular segment transverse, and width is significantly greater than length, gradually widen from base to end, each funicular segment with a round of short sensilla, clava clavate. Head in dorsal view 4.35× as broad as long, POL 2.3× OOL, without occipital margin (Fig. 4C). Head in lateral view, malar sulcus not distinct, malar space 0.44× eye height.

Mesosoma flattened, head and mesoscutum covered with distinct white setae and rough reticulation (Fig. 4F), notauli distinct and complete, pronotum 3.65× as long as broad, mesoscutum 2.95× as long as broad, notauli complete, scutulum 0.95× as long as broad, frenal line absent. Propodeum rectangular (Fig. 4G), plicae and median carina distinct and complete, white setae covered on both sides. Forewing hyaline (Fig. 4E), covered with dense setae, speculum absent, the distance from uncus to the postmarginal vein 0.63× the width of stigma, marginal vein 1.73× postmarginal vein, postmarginal vein longer than stigmal vein (2.8×).

Gaster sessile, ovate, 2.45× as long as broad, Gt_1_ covers 1/2 of gaster, ovipositor not exerted.

**Male.** Unknown.

##### Hosts.

Unknown.

##### Etymology.

‘*altunensis*’ means the collection site.

##### Diagnosis.

The new species is similar to *H.indica*, but noticeably different from *H.indica* in body color black with dark blue metallic reflection, head in frontal view as broad as height, pronotum 0.2× as long as broad, scutellum equal in length and width, and marginal vein: postmarginal vein: stigmal vein = 23:9.6:4.

### ﻿Pteromalidae

#### ﻿ Erdoesina maculata

Taxon classificationAnimaliaHymenoptera﻿Pteromalidae

Kang & Hu sp. nov.

F661CE6E-4C2F-5684-AD9B-F05041BB887D

https://zoobank.org/D94063EB-C71E-4780-93CC-A1FC9A3C31CF

Fig. 5A–G

##### Material examined.

***Holotype*.** • ♀, China, Xinjiang, Ruoqiang, Altun Mountain Nature Reserve, 37°48'14.2092"N, 89°54'41.2884"E, Altitude: 3449.93 m. 16.VII.2021. Coll. Ning Kang. by sweeping net. ***Paratypes*.** • 2 ♀♀, 1 ♂, 37°48'14.2092"N, 89°54'41.2884"E, Altitude:3449.93 m. 12.VII.2021. (All deposited in ICXU).

##### Description.

**Figure 5.**
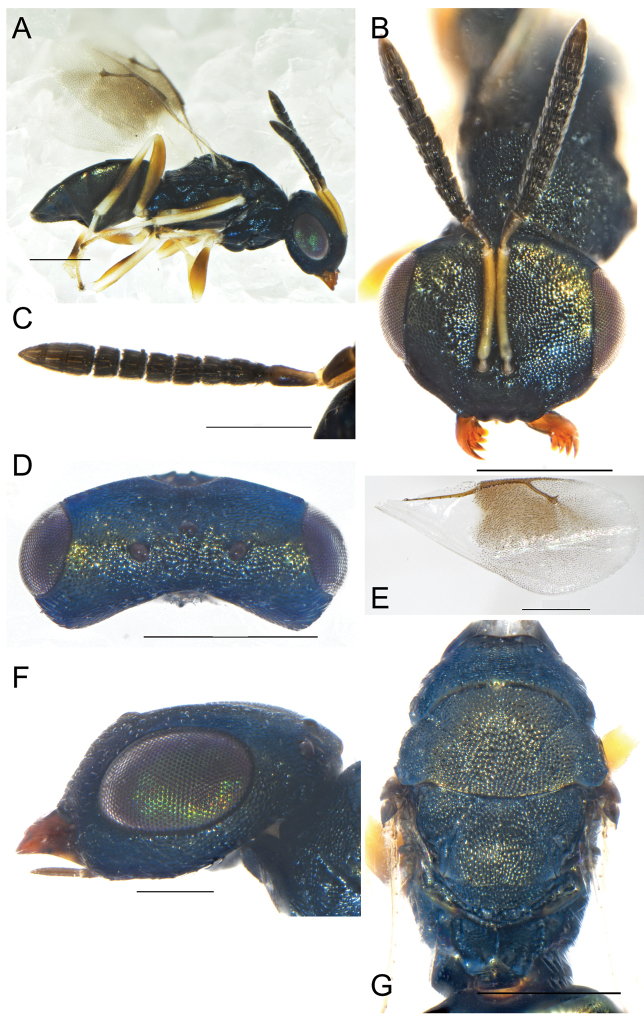
**A–G***Erdoesinamaculata* Kang & Hu, sp. nov., female holotype **A** habitus, lateral view **B** head, frontal view **C** antenna **D** head, dorsal view **E** forewing **F** head, lateral view **G** mesoscutum. Scale bars: 500 μm (**A, B, D, E, G**); 200 μm (**C, F**).

**Female.** Length 2.2 mm (Fig. 5A), body dark blue, eyes and ocelli dark red with purple metallic reflections, antennal scape light yellow, pedicel brown, flagellum dark brown; legs yellow except tibiae ends white, forewing hyaline with darkened area below the marginal and stigmal vein, venation brownish.

Head in frontal view 1.45× as broad as high (Fig. 5B), lower margin of clypeal curved, malar sulcus not distinct, mandible with four teeth. Antennal insertion on lower ocular line (Fig. 5C), antennal scape length 1.1× eye height, pedicel longer than F1, in lateral view 1.86× as long as broad, funicular segments slightly broader than length, clava longer than the combined length of last two funicles, length of flagellum and pedicel combined longer than head width (1.1×). Head in dorsal view (Fig. 5D), POL 1.54× OOL; Head in lateral view (Fig. 5F), malar sulcus absent, malar space 0.36× eye height.

Mesosoma slightly convex, pronotum short, mesoscutum 2.07× as broad as long, notaular lines shallow and incomplete, scutellum 1.48× as broad as long, frenal line not distinct (Fig. 5G). Propodeum smooth, median carina and plicae raised and complete; nucha short, rectangular smooth without reticulation. Forewing infuscate below the marginal vein and stigmal vein (Fig. 5E), speculum large and open below, basal cell bares except a few hairs apically, marginal vein 1.43× postmarginal vein, postmarginal vein 1.1× as long as the stigmal vein.

Gaster petiole short rectangular, slightly longer than thorax, the length of each segment uniform, the ovipositor does not protrude significantly from the end of the abdomen.

**Male.** Length 2 mm, body smaller, gaster flat, antennal flagellum with whorled finer setae.

##### Hosts.

Unknown.

##### Etymology.

“*maculata*” means dark spot, signifying the dark area of the female forewings.

##### Diagnosis.

The genus includes two species (Noyes, 2019): *E.alboannulata* mainly found in Europe and Central Asia, which has distinctly enlarged femora, gaster rounded, forewing marginal vein 1.5× as long as the stigmal vein, and basal cell bare; and *E.boarmiae* which has the marginal vein 1.3× as long as the stigmal vein, ovipositor slightly protruding from the end of the abdomen, head in dorsal view POL 1.05× OOL, and the combined length of two annuli and F1 as long as scape. The morphology of the new species is significantly different from the above two species.

#### ﻿ Homoporus flavus

Taxon classificationAnimaliaHymenoptera﻿Pteromalidae

Kang & Hu sp. nov.

7409E25F-D8D6-5FD6-885A-4273B498154A

https://zoobank.org/A118D174-FBD9-4503-AAA6-ED7D503CB84A

Fig. 6A–G

##### Material examined.

***Holotype*.** • ♀, China, Xinjiang, Ruoqiang, Altun Mountain Nature Reserve, 37°18'21.9924"N, 90°20'33.2592"E, Altitude: 3907.8 m, 7.VII.2019. Coll. Ning Kang, by sweeping net. ***Paratypes*.** • 1 ♀1 ♂, 37°23'51.6516"N, 90°10'59.7216"E, Altitude: 3855.19 m, 7.VII.2019. Coll. Ning Kang, by sweeping. • 2 ♀ 1 ♂, 37°23'49.056"N, 90°10'52.3092"E, Altitude: 3854.2 m, 2020.VII.18. • 1 ♀ 2 ♂, 37°23'53.0664"N, 90°11'11.8644"E, Altitude: 3843.19 m, 2021.VII.14 (All deposited in ICXU).

##### Description.

**Figures 6.**
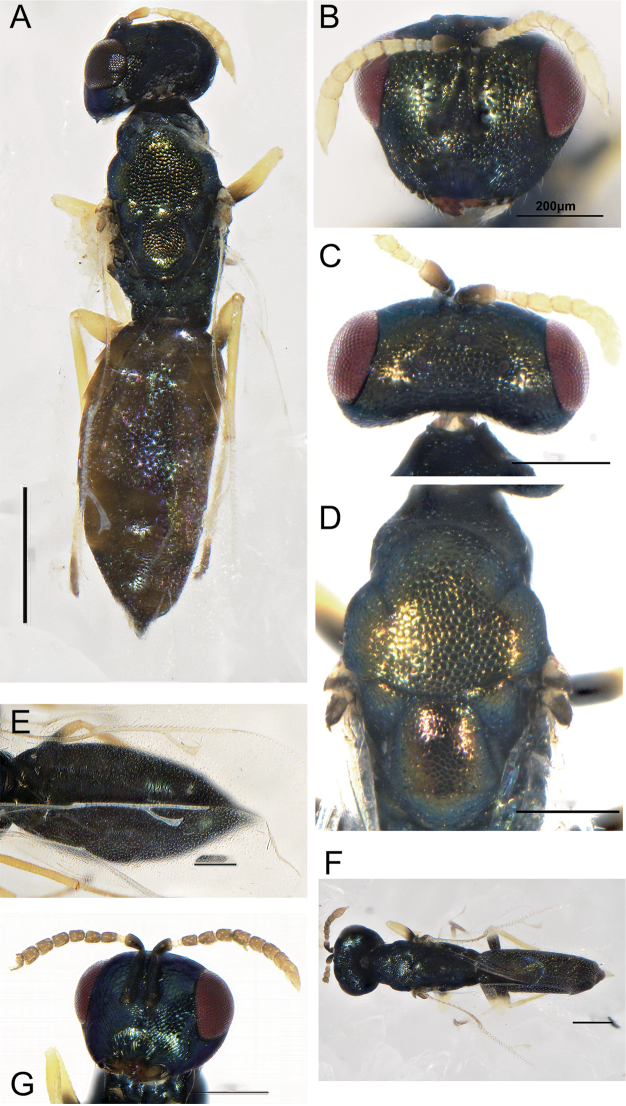
**A–G***Homoporusflavus* Kang & Hu, sp. nov., female, holotype **A** habitus, dorsal view **B** head, frontal view **C** head, dorsal view **D** mesoscutum, dorsal view **E** forewing **F** male, habitus **G** head, frontal view. Scale bars: 500 μm (**A**); 200 μm (**B−G**).

**Female.** Length 2.2 mm, body slender, head and thorax dark green, with metallic reflection, gaster dark brown (Fig. 6A); eyes and ocelli dark red, mandible yellowish-brown; antennal flagellum light yellow, scape dark green with metallic reflection, pedicel dark brown; coxae concolor with body, legs yellow except basal part of femurs dark brown; forewing hyaline, venation albescent.

Head in frontal view 1.3× as wide as high (Fig. 6B), covered with rough reticulate, clypeal margin straight, clypeus with longitudinal striation. Antennal insertion on lower ocular line, distance from upper margin of torulus to lower margin of anterior ocellus 1.6× distance from lower margin of torulus to lower margin of clypeus. Antennal scape not reaching vertex, two discoid anelli, F1 shortest and square, clava 2.55× as long as wide, and with tubercles at apical, length of flagellum and pedicel combined longer than head width (0.86×). Head in lateral view, malar sulcus slender not distinct, eyes height 1.62× malar space. Head in dorsal view (Fig. 6C), 2.45× as wide as height, POL 1.84× OOL.

Mesosoma flattened (Fig. 6D), pronotum without carina, significantly narrower than the mesoscutum, mesoscutum 1.22× as wide as long, notauli incomplete, scutulum 1.32× as wide as long, frenal line not distinct, propodeum with finer reticulate in the middle area, median carina and plica not distinct, nucha short. Forewing hyaline (Fig. 6E) marginal vein 1.28× postmarginal vein, postmarginal vein longer than stigmal vein (1.38×), basal cell bare, speculum small.

Gaster sessile, flattened, 2.43× as long as wide, slightly longer than the combined length of head and thorax, ovipositor does not protrude.

**Male.** Length 1.7 ± 0.1 mm (Fig. 6F), body slender, head and thorax dark blue, gaster dark brown, forewing hyaline, venation light brown, antennal pedicel dark brown, anellus light yellow, flagellum brown, each funicular segments with whorled light seta (Fig. 6G).

##### Hosts.

Unknown.

##### Etymology.

‘*flavus*’ means golden yellow, which is the distinctive color of its antennae.

##### Diagnosis.

The new species is similar to *H.sinensis*, but differs in having POL 1-1.11× OOL, each funicular segment longer than broad, marginal vein 2.3× stigmal vein, and mesosoma 2× as long as broad.

#### ﻿ Stenomalina viridis

Taxon classificationAnimaliaHymenoptera﻿Pteromalidae

Kang & Hu sp. nov.

BA4D3F51-86D4-56CC-BFB9-F8A378D01116

https://zoobank.org/D2946720-B2EC-4E63-8785-327CEBA78DFF

Fig. 7A–F

##### Material examined.

***Holotype*.** • ♀, China, Xinjiang, Ruoqiang, Altun Mountain Nature Reserve, 36°58'10.8984"N, 90°14'44.916"E, Altitude: 4021.95 m, 21.VII.2020. Coll. Ning Kang, by sweeping net. ***Paratypes*.** • 4 ♀2 ♂, 36°56'25.8576"N, 90°16'48.2376"E, Altitude: 4052.62 m, 21.VII.2020. Coll. Ning Kang, by sweeping. • 2 ♀ 1 ♂, 36°58'10.8984"N, 90°14'44.916"E, Altitude: 4021.95 m, 16.VII.2020. Coll. Ning Kang, by sweeping. • 50 ♀, 36°56'32.91"N, 90°16'40.4508"E, Altitude: 4047.73 m, 16.VII.2021. Coll. Ning Kang, by sweeping.

##### Description.

**Figures 7.**
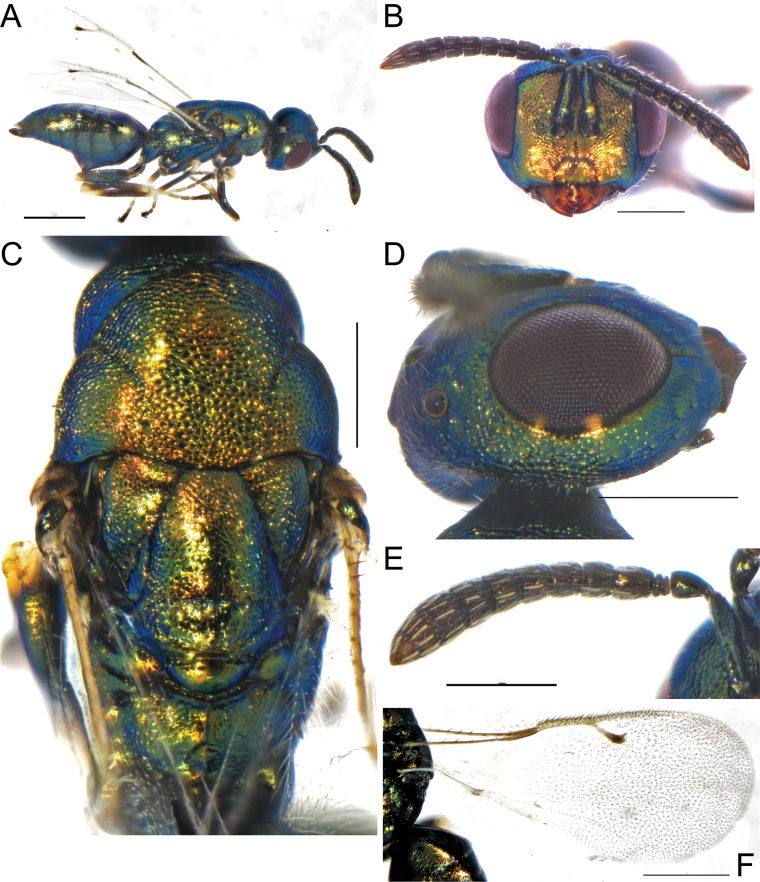
**A–F***Stenomalinaviridis* Kang & Hu, sp. nov., female holotype **A** habitus, dorsal view **B** head, frontal view **C** mesoscutum, dorsal view **D** head, lateral view **E** antenna **F** forewing. Scale bars: 500 μm (**A, F**); 250 μm (**B–E**).

**Female.** Body length 2.3 mm (Fig. 7A), body green with metallic luster, eyes and ocelli dark red, scape and pedicel concolor with body, flagellum dark brown, femur dark brown except light yellow at both ends, forewing hyaline, venation brown.

Head in frontal view 1.4× as wide as high (Fig. 7B), clypeal suture obvious, clypeus with longitudinal striation, clypeal margin protruded with three teeth, middle tooth sharp and long, side teeth shorter. Antennal insertion above lower ocular line, distance from upper margin of torulus to lower margin of anterior ocellus 1.55× distance from lower margin of torulus to lower margin of clypeus, scape length 0.65× eye height, not reaching anterior ocellus, pedicel pyriform (Fig. 7E), 1.65× as long as broad, as long as F1, two discoid anelli, funicular segments square, and gradually widen towards the end, calva 2.25× as long as broad, each funicular segment with sensilla, length of flagellum and pedicel combined longer than head width (1.08×). Head in lateral view (Fig. 7D), malar sulcus distinct, malar space 0.4× eye height. Head in dorsal view, POL 1.63× OOL.

Mesosoma slender (Fig. 7C), pronotum anteriorly not margined, notauli incomplete, mesoscutum 1.49× as long as broad, covered with rough reticulation, scutellum slightly convex, 1.02× as long as broad, frenal line strongly impressed, reticulation of frenum similar to the scutellum, propodeum length 0.46× as long as scutellum, median carina slim, plicae not distinct. Forewing costal cell with few setae apically, basal cell bare, speculum large and opened below, marginal vein 1.55× postmarginal vein, postmarginal vein 0.65× stigmal vein, stigma oblong with long uncus (Fig. 7F).

Gaster sessile and oval, slightly longer than thorax, Gt_1_ 0.35× length of gaster.

**Male.** Body length 2.1 ± 0.1 mm (Fig. 7F), flagellum with whorled setae.

##### Hosts.

Unknown.

##### Etymology.

‘*viridis*’ means emerald green, emphasizing its distinctive body color.

##### Diagnosis.

The new species is similar to *S.muscarum* (L.), but can be differentiated by the pronotum anteriorly margined, forewing marginal vein 2.2–2.5× stigmal vein, and male flagellum black with metallic luster.

#### ﻿ Thinodytes splendens

Taxon classificationAnimaliaHymenoptera﻿Pteromalidae

Kang & Hu sp. nov.

8848D134-EFBC-5059-AF81-B15F6744E4CA

https://zoobank.org/F62377E7-873A-4D38-AFA4-94DA6AF318AB

Fig. 8A–G

##### Material examined.

***Holotype*.** • ♀, China, Xinjiang, Ruoqiang, Altun Mountain Nature Reserve, 37°18'21.9924"N, 90°20'33.2592"E, Altitude: 3907.8 m, 7.VII.2019. Coll. Ning Kang, by sweeping net. ***Paratypes*.** • 1 ♀ 1 ♂, 37°23'51.6516"N, 90°10'59.7216"E, Altitude: 3855.19 m, 7.VII.2019. Coll. Ning Kang, by sweeping net.

##### Description.

**Figures 8.**
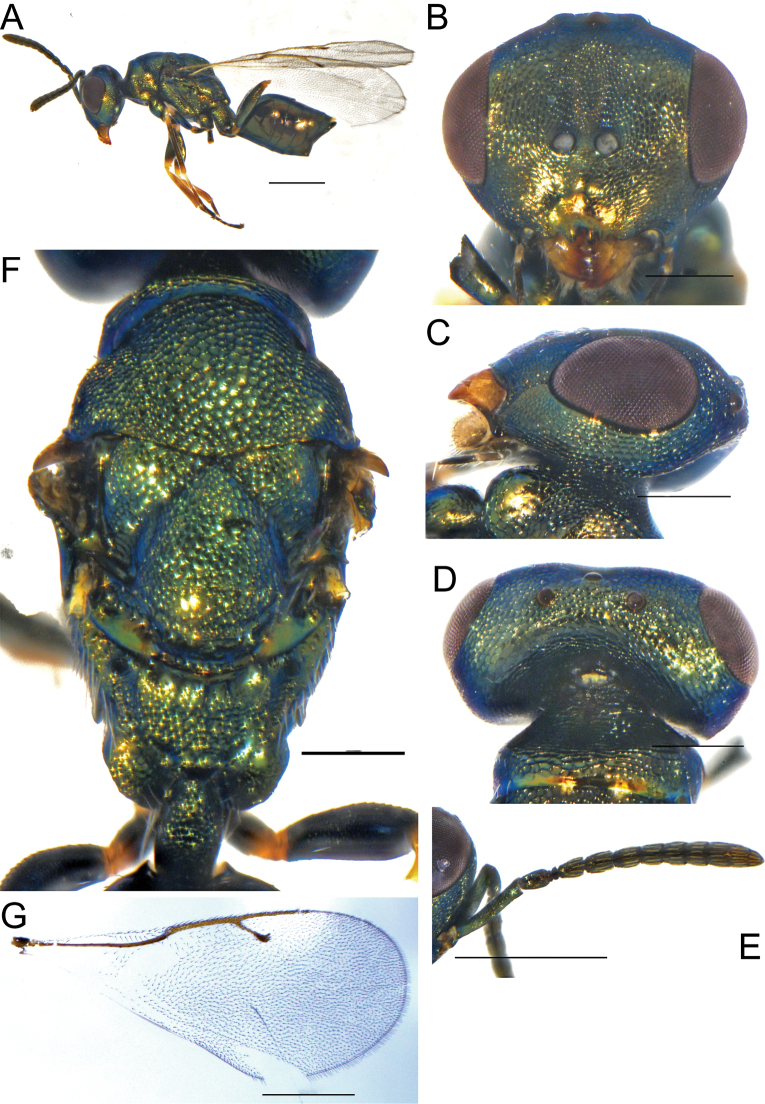
**A–G***Thinodytessplendens* Kang & Hu, sp. nov., female holotype **A** habitus, lateral view **B** head, frontal view **C** head, lateral view **D** head, dorsal view **E** antenna **F** mesoscutum **G** forewing. Scale bars: 500 μm (**A, G, E**); 200 μm (**B−D**).

**Female.** Length 2.2 mm (Fig. 8A), body dark green with metallic reflection; eyes dark red, mandible dark brown; flagellum dark brown, scape and pedicel concolor with body; tibia brown, tarsus dark brown; forewing hyaline, venation brown.

Head in frontal view 1.53× as long as wide (Fig. 8B), eyes height 2.75× malar space, clypeus smooth, anterior margin of clypeus with three asymmetrical teeth, mandible four teeth. Antennae inserted above level of ventral edge of eyes, formula 11263, scape 0.95× as long as eye height, length of flagellum and pedicel combined longer than head width (1.2×), anterior four funicular segments 1.63× as long as broad, the last two funicular segments 1.15× as long as broad, clava with micropilosity area at the ventral of last two segments (Fig. 8E), and slightly shorter than last two funicular segments. Head in dorsal view (Fig. 8D), POL 1.45× OOL. Head in lateral view (Fig. 8C), malar sulcus not distinct, malar space 0.36× eyes height.

Pronotum covered with large reticulates (Fig. 8F), mesoscutum convex, 2.05× as broad as long, notauli complete but shallow, scutellum 1.33 as broad as long, frenal line not distinct, reticulations on scutellum smaller than mesoscutum, frenum smooth without reticulates. Propodeum as long as scutellum, median carina and plica not distinct, each side of the upper margin with elliptical depression, callus covered with white dense setae. Forewing with 2–3 rows hairs on the end of costal cell (Fig. 8G), 4 rows of setae in the basal cell, speculum oval and closed below, marginal vein as long as postmarginal vein, 2× as long as stigmal vein.

Gaster petiole slightly longer than propodeum, 1.93× as long as wide, abdomen oval-shaped and significantly shorter than the thorax, 0.63× as long as thorax. Gt_1_ 0.48× as long as gaster.

**Male.** Length 1.8 ± 0.1 mm, gaster flat and short, flagellum covered with dense setae.

##### Hosts.

Unknown.

##### Etymology.

‘*splendens*’ means luminous, emphasizing its distinctive bright-green body color among the species in the genus.

##### Diagnosis.

By comparing the morphology with the seven species of this genus that have been described in the world (Heydon 1995), it was found that the new species is most similar to *T.cyzicu*, but the body color of the new species is obviously bright green, while the body color of the latter species is blue-black, the head in frontal view is 1.25× as long as wide, the propodeum has a complete median carina and plica, and the spiracular sulcus wide and deep.

### ﻿Eulophidae

#### ﻿ Diaulinopsis altunensis

Taxon classificationAnimaliaHymenoptera﻿Eulophidae

Kang & Hu sp. nov.

5804763C-67DE-58BE-A4B3-BB0D04CD16A8

https://zoobank.org/70983ACC-A8BD-40F3-9F4F-7DBF74904647

Fig. 9A–G

##### Material examined.

***Holotype*.** • ♀, China, Xinjiang, Ruoqiang, Altun Mountain Nature Reserve, 38°5'37.572"N, 89°16'17.1372"E, Altitude: 3471.91 m, 15.VII.2020. Coll. Ning Kang, by sweeping net. ***Paratypes*.** • 1 ♀ 2 ♂, 37°49'28.8588"N, 89°9'9.5328"E, Altitude: 4237.16 m, 12.VII.2020. Coll. Ning Kang, by sweeping; • 1 ♂, 37°48'14.2092"N, 89°54'41.2884"E, Altitude: 3449.93 m, 12.VII.2020, by net sweeping; • 1 ♂, 37°39'47.6172"N, 88°45'35.1864"E, H: 4007.46 m, 13.VII.2020, • 2 ♀, 37°58'30.1512"N, 88°58'25.158"E, Altitude: 3849.56 m, 14.VII.2020; • 4 ♀ 2 ♂, 38°5'37.572"N, 89°16'17.1372"E, Altitude: 3471.91 m, 9–15.VII.2020, by Malaise trap; • 6 ♂, 36°56'32.91"N, 90°16'40.4508"E, H: 4047.73 m, 16.VII.2020, by net sweeping.

##### Description.

**Figures 9.**
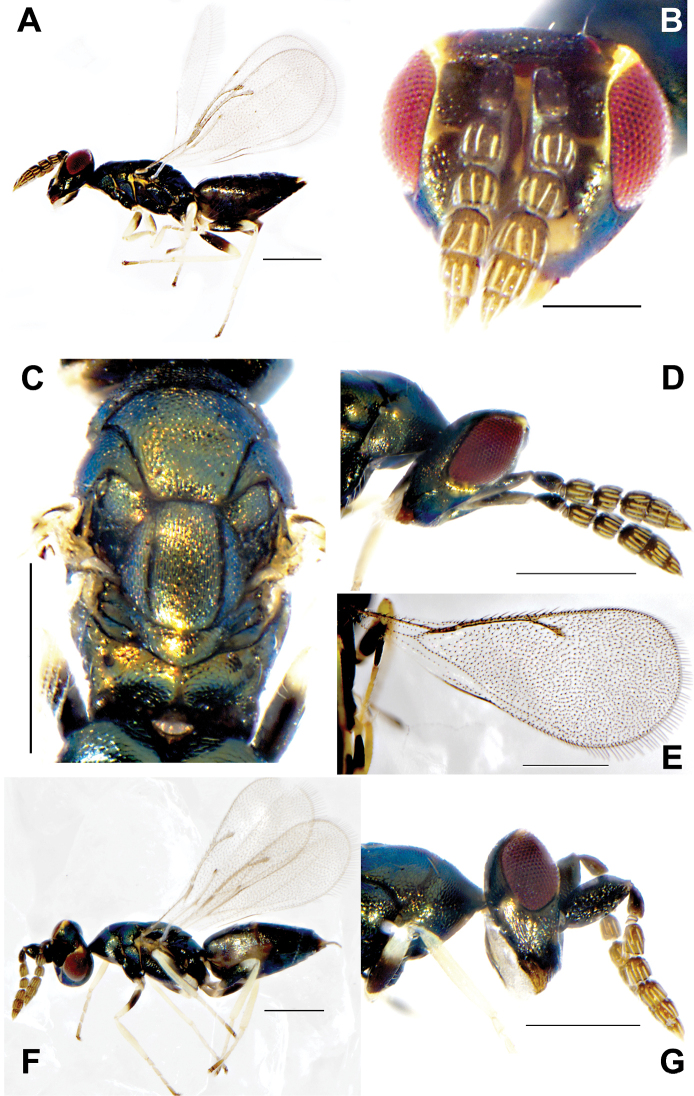
**A–G***Diaulinopsisaltunensis* Kang & Hu, sp. nov., female holotype **A** habitus, lateral view **B** head, frontal view **C** mesoscutum **D** head, lateral view **E** forewing. Male **F** habitus, lateral view **G** head, lateral view. Scale bars: 250 μm (**A, C, D−G**); 100 μm (**B**).

**Female.** Length 1.4 mm, body metallic green (Fig. 9A), antenna dark brown except scape complete brown with metallic tints, anterior of eyes, toruli and part of frontal suture with some yellowish marks, eyes and ocellus red, mandibles yellow and maxillary palp white without expansion; legs yellowish-white, coxae with body color, basal 3/4 of femur dark brown with metallic tints, terminal tarsus brown. Gaster dark brown with metallic green tints.

Head with finely reticulate, in frontal view 1.05× as wide as high (Fig. 9B), POL: OOL = 3.65. Frontal sulcus obtuse “V” shaped and yellow marked, malar sulcus straight, 0.4× as eye height; clypeus truncate, without oral fossa. Antenna inserted below the level of lower margin of eyes, with two discoid anelli (Fig. 9D); scape 4.5× as long as wide, 0.8× as long as eye height; clava 2.4× as long as wide and 1.4× as long as F1 + F2, F1 1.3× as wide as the width of pedicel in lateral view; funiculars quadrate, each with a single row of sensilla and irregularly distributed.

Mesosoma, pronotum conoid (Fig. 9C), 0.2× as long as wide, mesoscutum 0.8× as long as wide, with complete and deep notauli; scutellum 1.0× as long as wide and 1.0× as long as mesoscutum, with lateral grooves; ratio distance between grooves/distance between one groove and lateral margin of scutellum 2.3, meshes of reticulation more elongate than mesoscutum; dorsellum 0.2× as long as scutellum, propodeum 0.2× as long as scutellum, smooth with fine reticulation, propodeal callus with four pale setae. Submarginal vein of forewing with six setae and without speculum (Fig. 9E), marginal vein: postmarginal vein: stigmal vein = 3:1.7:1.

Metasoma, gaster sessile, dark brown with metallic tints throughout, about 1.7–1.8× as long as wide.

**Male.** Length 1.7 ± 0.1 mm (Fig. 9F), body slighter, antennal scape obviously enlarged (Fig. 9G).

##### Hosts.

Unknown. There are many Agromyzidae (Diptera) flies and *Oxytropis*, *Carex* plants (gravel desert) in their habits.

##### Etymology.

‘*altunensis*’ means the collection site.

##### Diagnosis.

The new species is similar to *D.albimaxillia*, but can be distinguished by the significantly enlarged white maxillary palp and gaster with a large spot in the anterior 1/2 of the latter. Among species of the genera, only this species has the scape and gaster dark brown with metallic green tints throughout.

#### ﻿ Hyssopus altunensis

Taxon classificationAnimaliaHymenoptera﻿Eulophidae

Kang & Hu sp. nov.

A9ED9018-AF37-5F4D-ACE2-257DA83EBF39

https://zoobank.org/CD4F004E-9024-47EE-B5DB-12F67F6E3A65

Fig. 10A–I

##### Material examined.

***Holotype*.** • ♀, China, Xinjiang, Ruoqiang, Altun Mountain Nature Reserve, 37°18'21.9924"N, 90°20'33.2592"E, Altitude: 3907.8 m, 7.VII.2019. Coll. Ning Kang, by sweeping net. ***Paratypes*.** • 1 ♀ 1 ♂, 37°23'51.6516"N, 90°10'59.7216"E, Altitude: 3855.19 m, 7.VII.2019. Coll. Ning Kang, by sweeping.

##### Description.

**Figures 10.**
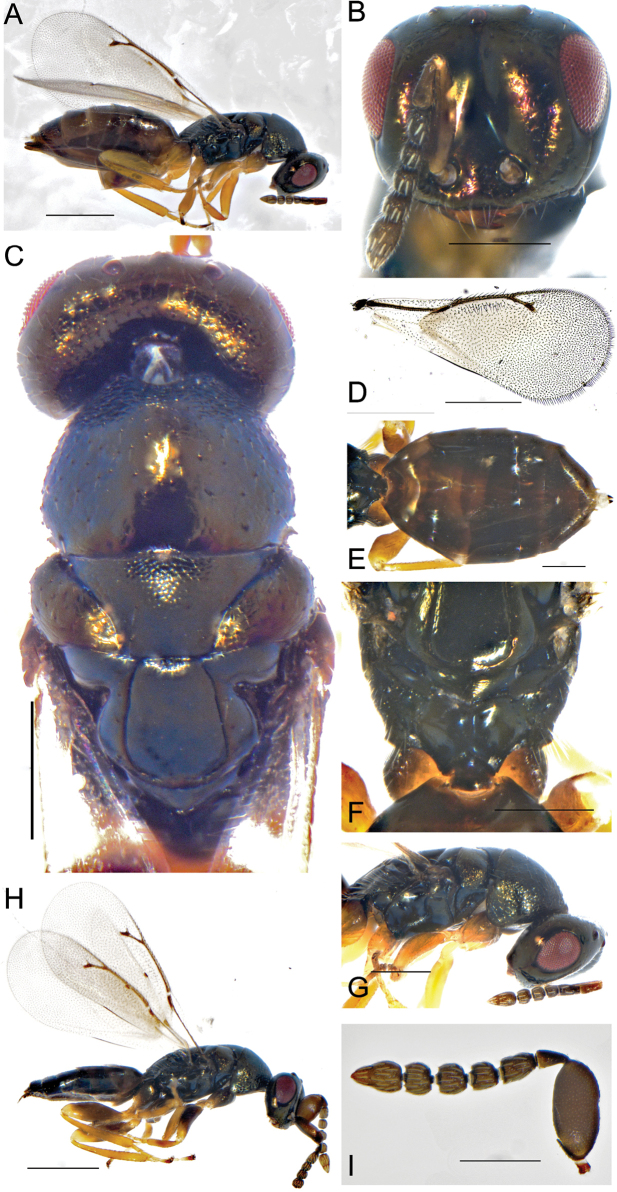
**A–I***Hyssopusaltunensis* Kang & Hu, sp. nov., female holotype **A** habitus, lateral view **B** head, frontal view **C** mesoscutum **D** forewing. Male **E** habitus, dorsal view **F** forewing **G** head, mesoscutum, lateral view **H** male, habitus, lateral view **I** antenna. Scale bars: 500 μm (**A, D, H**); 200 μm (**B, C, E−G, I**).

**Female.** Length 2.4 mm, body and thorax dark green, with metallic reflection, gaster dark brown (Fig. 10A), antennae brown, eyes red; coxae yellowish-brown, distal tarsus dark brown; forewing hyaline, tegula brown, venation brown.

Head in frontal view square (Fig. 10B), 1.25× as wide as high, scape 1.85× as long as eyes, clypeus margin 3.25× malar space, clypeus with long white hair; antennal insertion close to clypeus margin, below the lower ocular line; pedicel longer than each funicular, 2.34×, anelli short and transverse, F1 longer, F2-F4 0.88× as long as wide, each funicular with 2 row of sensilla, clava clavate, with a strong constriction between its second and third segments, 1.95× as long as wide. Head in lateral view (Fig. 10G), eyes 1.55× as height as wide, malar sulcus straight, malar space 1.65× as long as eyes height. Head in dorsal view, 4.25× as wide as height, POL 2.15× OOL.

Mesoscutum not distinctly convex (Fig. 10C), smooth with fine reticulation, pronotum large and hemisphere, covered with irregular setae, 1.3× as long as mesoscutum; mesoscutum smooth with 2 pairs of setae, 2.85× as wide as long, without setae; scutulum 1.25× as long as mesoscutum, sublateral groove connected terminally, frenum smooth without reticulation, significantly shorter than propodeum; propodeum with complete median carina (Fig. 10F), without plica and costulae, nucha short. Forewing hyaline (Fig. 10D), 2.26× as long as wide, marginal cell with 2–3 rows of setae, speculum not distinct.

Gaster 1.95× as long as wide (Fig. 10E), full oval shape, ovipositor sheath slightly protruding the end of the abdomen.

**Male.** Length 1.8 ± 0.1 mm (*N* = 5) (Fig. 10H), antennal scape significantly expands, 1.5–1.6× as long as wide (Fig. 10I); F1 with at least 2 rows of sensilla, F2-4 with 1 row of irregular setae; each coxa concolor with body, femur dark brown; gaster flattened and shorter than thorax.

##### Hosts.

Unknown.

##### Diagnosis.

Comparing the morphological characters of the genus *Hyssopus* (Schauff, 1985), the new species is similar to *Hyssopusgeniculatus* (Hartig, 1838), but the latter has a body black, eyes black, ocellus brown, each coxa black, antennal insertion on lower ocular line, and the flagellum is widened gradually towards the end. The new species differs from *Hyssopusflavgasterus* by the body dark green, with blue luster, eyes dark gray, and antennal insertion below the lower ocular line.

Geographical distribution of Eunotidae, Herbertiidae, Pteromalidae and Eulophidae in the Altun Mountain National Nature Reserve

The Altun Mountain region exhibits pronounced topographic and vegetative contrasts between its eastern and western sectors. The eastern foothills are characterized by gentle terrain with verdant vegetation, while the western alpine zone demonstrates rugged topography and harsher habitat conditions. We systematically examined the east-west disparity in the four families’ species diversity and community structure.

The distribution of the four families across various habitats within the Altun Mountain Nature Reserve is extensive but notably uneven. Species abundance and population density are significantly higher in the eastern part of the reserve compared to the western part, while both population numbers and species richness are lower. The eastern sector of the nature reserve exhibits higher vegetation richness and coverage compared to the western region, with a marginally elevated mean annual temperature that collectively is more favorable for species survival. Species of Eunotidae and Herbertiidae are mainly distributed in the western part of the reserve, having a typical psammophytic desert ecosystem characterized by arenaceous soil substrates (sand content > 85%), where xerophytic leguminous shrubs constitute 22−35% of the total vegetation cover, demonstrating significant adaptation to arid edaphic conditions. Among Pteromalidae, the subfamilies Miscogasterinae and Pteromalinae are widely distributed with substantial populations, while Eulophidae, Entedontinae and Eulophinae exhibit a similar distribution pattern. At the genus level, *Selderma*, *Pachyneuron*, *Callicarolynia*, *Halticoptera*, *Neochrysocharis*, *Entedon*, *Diaulinopsis* are notably prevalent throughout the reserve. The distributions of *Halticopteratrinflata* Huang, 1991 and *Callicarolyniayixiekea* Kang & Hu, 2022 were found to be clustered, with *H.trinflata* showing a strong association with *Thermopsisalpina* (Pall.) Ledeb. Additionally, we observed that sites with higher coverage of flowering vegetation had significantly greater species abundance.

## Supplementary Material

XML Treatment for Eunotus caeruleus

XML Treatment for Eunotus argenteus

XML Treatment for Herbertia altunensis

XML Treatment for Erdoesina maculata

XML Treatment for Homoporus flavus

XML Treatment for Stenomalina viridis

XML Treatment for Thinodytes splendens

XML Treatment for Diaulinopsis altunensis

XML Treatment for Hyssopus altunensis

## ﻿Additional information

### Author ORCIDs

Ning Kang https://orcid.org/0000-0002-9616-6886

Hong-Ying Hu https://orcid.org/0000-0002-2295-1072

### Data availability

All of the data that support the findings of this study are available in the main text.

## ﻿Appendix 1

**Checklist of Eunotidae, Herbertiidae, Pteromalidae and Eulophidae in Altun Mountain National Nature Reserve**

The checklist is the result of a three-year investigation in the Altun Mountain National Nature Reserve. The following 61 species of the four families were found:

**Eunotidae Ashmead, 1904**

1. *Eunotuscaeruleus* Kang & Hu, sp. nov.

2. *Eunotusargenteus* Kang & Hu, sp. nov.

**Herbertiidae Bouček, 1988**

3. *Herbertiaaltunensis* Kang & Hu, sp. nov.

**Pteromalidae Dalman, 1820**

**Asaphinae Ashmead, 1904**

4. *Asaphespetiolatus* (Zetterstedt, 1838)

5. *Asaphesvulgaris* Walker, 1834

**Miscogasterinae Walker, 1833**

**Miscogastrini**:

6. *Seladermasaurus* Walker, 1844

7. *Xestomnastermazares* (Walker, 1844)

**Sphegigastrini**:

8. *Ammeiapulchella* Delucchi, 1962

9. *Sphaeripalpusvulgaris* Huang, 1990

10. *Callicarolyniayixiekea* Kang & Hu, 2023

11. *Cyrtogastervulgaris* Walker, 1833

12. *Sphegigasterintersita* (Graham, 1969)

13. *Halticopteratrinflata* Huang, 1991

14. *Halticopteramoczari* (Erdös, 1954)

15. *Halticopteracircula* Walker, 1833

16. *Halticopteraatherigona* (Huang, 1990)

17. *Merismussplendens* Graham, 1969

18. *Thinodytessplendens* Kang & Hu, sp. nov.

**Pireninae Haliday, 1844**

19. *Gastrancistrushamillus* Graham, 1969

**Pachyneurinae Rohdendorf, 1962**

20. *Pachyneuronaphidis* (Bouché, 1834)

21. *Pachyneuronsolitarium* (Hartig, 1838)

22. *Pachyneuronkorlense* Xiao, Jiao & Huang, 2009

23. *Pachyneurongrande* Thomson, 1878

24. *Pachyneuronplaniscuta* Thomson, 1878

**Pteromalinae Dalman, 1820**

**Pteromalini**:

25. *Chlorocytussplendensis* Li & Hu, 2017

26. *Chlorocytuspolichna* (Walker, 1848)

27. *Caenocrepisarenicola* (Thomson, 1878)

28. *Cyclogastrellaqinghaiensis* Jiao & Xiao, 2015

29. Dibrachys (Dibrachys) microgastri (Bouché, 1834)

30. *Erdoesinamaculata* Kang & Hu, sp. nov.

31. *Homoporusflavus Kang* & Hu, sp. nov.

32. *Mesopolobussemenis* Askew, 1997

33. *Mesopolobusbruchophagi* (Gahan, 1917)

34. *Nasoniavitripennis* (Walker, 1836)

35. *Pteromalusmicrops* (Graham, 1969)

36. *Pteromalussequester* Walker, 1835

37. *Stenomalinaviridis* Kang & Hu, sp. nov.

38. *Trichomalopsisbrevis* Kang & Hu, sp. nov.

39. *Trichomalopsisclosterae* Kamijo, 1983

40. *Urolepismaritima* (Walker, 1834)

**Eulophidae Westwood, 1829**:

**Entedoninae Förster, 1856**

41. *Chrysocharismediana* Förster, 1861

42. *Entedoncyanellus* Dalman, 1820

43. *Neochrysocharisaratus* (Walker, 1838)

44. *Neochrysocharisformosus* (Westwood, 1833)

45. *Neochrysocharisiris* Erdös, 1954

**Eulophinae Westwood, 1829**

46. *Cirrospilusvittatus* Walker, 1838

47. *Diaulinopsisaltunensis* Kang & Hu, sp. nov.

48. *Diglyphusisaea* (Walker, 1838)

49. *Diglyphusbegini* (Ashmead, 1904)

50. *Elachertusparvispecularis* Zhu & Huang, 2001

51. *Elachertuslateralis* (Spinola, 1808)

52. *Hyssopusaltunensis* Kang & Hu, sp. nov.

**Tetrastichinae Förster, 1856**

53. Aprostocetus (A.) eurystoma Graham, 1961

54. Aprostocetus (A.) emesa (Walker, 1839)

55. Aprostocetus (Chrysotetrastichus) celtidis (Walker, 1839)

56. Aprostocetus (A.) caudatus Westwood, 1833

57. *Baryscapusfossarum* Graham, 1991

58. *Baryscapusgradwelli* Graham, 1991

59. *Holcotetrastichusrhosaces* (Walker, 1839)

60. *Kolopternakohatensis* Graham, 1987

61. *Oomyzussokolowskii* (Kurdjumov, 1912)

## References

[B1] Bhat DM Bhagat RC Qureshi AA (2017) Parasitoid fauna associated with insect pests of vegetable crops of Kashmir Himalaya, India: Check List and Biodiversity. Munis Entomology and Zoology 12: 168–174.

[B2] Bouček Z (1988) Australasian Chalcidoidea (Hymenoptera). A biosystematic revision of genera of fourteen families, with a reclassification of species. CAB International; Aberystwyth, Wales: The Cambrian News Ltd, Wallingford, UK, 832 pp.

[B3] Bouček Z Rasplus JY (1991) Illustrated key to West-Palearctic genera of Pteromalidae. Institut National de la Recherche Agronomique, Paris, 140 pp.

[B4] Burks R Mitroiu M-D Fusu L Heraty JM Janšta P Heydon S Papilloud ND-S Peters RS Tselikh EV Woolley JB van Noort S Baur H Cruaud A Darling C Haas M Hanson P Krogmann L Rasplus J-Y (2022) From hell’s heart I stab at thee! A determined approach towards a monophyletic Pteromalidae and reclassification of Chalcidoidea (Hymenoptera). Journal of Hymenoptera Research 94: 13–88. 10.3897/jhr.94.94263

[B5] Cruaud A Rasplus J-Y Zhang J Burks R Delvare G Fusu L Gumovsky A Huber JT Janšta P Mitroiu M-D Noyes JS Van Noort S Baker A Böhmová J Baur H Blaimer BB Brady SG Bubeníková K Chartois M Copeland RS Papilloud ND-S Dal Molin A Dominguez C Gebiola M Guerrieri E Kresslein RL Krogmann L Lemmon EM Murray EA Nidelet S Nieves-Aldrey JL Perry RK Peters RS Polaszek A Sauné L Torréns J Triapitsyn S Tselikh EV Yoder M Lemmon AR Woolley JB Heraty JM (2022) The Chalcidoidea bush of life – a massive radiation blurred by mutational saturation. Evolutionary Biology. 10.1101/2022.09.11.507458

[B6] Dzhanokmen KA (2017) Pteromalids (Hymenoptera, Chalcidoidea: Pteromalidae) of the Karatau Ridge and adjacent territories of the Talas Alatau Ridge in Western Tien Shan. Entomological Review 97: 794–817. 10.1134/S0013873817060082

[B7] Gibson GAP Huber JT Woolley JB (1997) Annotated keys to the genera of Nearctic Chalcidoidea (Hymenoptera). National Research Council Research Press, Ottawa, Canada, 327–429.

[B8] Graham MWRV (1969) The Pteromalidae of North-western Europe (Hymenoptera: Chalcidoidea). Bulletin of the British Museum (Natural History), London, 908 pp. 10.5962/p.258046

[B9] Hannes B Hansson C (1997) *Chrysocharisfrigida* n. sp., a new Holarctic species of Entedoninae (Hymenoptera: Eulophidae). Bulletin de laSociete Entomologique suisse 70: 203–207.

[B10] Heydon SL (1995) A Review of the North American Species of *Thinodytes* Graham and *Mauleus* Graham (Hymenoptera: Pteromalidae). Journal of Hymenoptera Research 4: 1–24.

[B11] Huang DW Xiao H (2005) Fauna Sinica, Insecta, Volume 42, Hymenoptera: Pteromalidae. Science press, Beijing, China, 388 pp.

[B12] Japoshvili G Kostjukov VV (2016) The first record of *Pronotalia* Gradwell, 1957 (Hymenoptera, Eulophidae) Wasps from Georgia. Entomological Review 96: 1089–1091. 10.1134/S0013873816080121

[B13] Kang N Hu H Huang Z Luo S Guo S (2023) Environmental factors drive chalcid body size Increases with altitudinal gradients for two hyper-diverse taxa. Insects 14: 67. 10.3390/insects14010067 PMC9865982 36661995

[B14] Kevan PG (1973) Parasitoid wasps as flower visitors in the Canadian high arctic. Anzeiger für Schädlingskunde Pflanzen- und Umweltschutz 46: 3–7. 10.1007/BF01992960

[B15] Khan MA (1984) Two new species of *Chrysocharis* Forster (Hymenoptera;Eulophidae) from high altitude of India. Journal Bombay Natural History Society 82: 376–378.

[B16] Kumar R Sharma PL (2016) Studies on diversity and abundance of parasitoids of *Chromatomyiahorticola* (Goureau) (Agromyzidae: Diptera) in north-western Himalayas, India. Journal of Applied and Natural Science 8: 2256–2261. 10.31018/jans.v8i4.1121

[B17] Noyes JS (2019) Universal Chalcidoidea Database. Universal Chalcidoidea Database. https://www.nhm.ac.uk/our-science/data/chalcidoids/database/ [Accessed 10 November 2025]

[B18] Schauff M (1985) Revision of the Nearctic species of *Hyssopus* Girault (Hymenoptera: Eulophidae). Journal of the New York Entomological Society 93: 1096–1108.

[B19] Sha Z-L Zhu C-D Murphy RW Huang D-W (2007) *Diglyphusisaea* (Hymenoptera: Eulophidae): a probable complex of cryptic species that forms an important biological control agent of agromyzid leaf miners. Journal of Zoological Systematics and Evolutionary Research 45: 128–135. 10.1111/j.1439-0469.2006.00375.x

[B20] Sureshan PM (2012) New Distributional Records of Pteromalidae (Hymenoptera: Chalcidoidea) from Ladakh, India. Records of the Zoological Survey of India 112: 39. 10.26515/rzsi/v112/i4/2012/122017

[B21] Trjapitzin VA (1989) Parasitic Hymenoptera of the Fam. Encyrtidae of Palaearctics. Opredeliteli po Faune SSSR 158: 332.

[B22] Xiao H Huang D (2004) Preliminary study of Pteromalid (Hymenoptera, Pteromalidae) diversity in Qinghai Xizang plateau, taxonomy and biogeography. Acta Zootaxonomica Sinica 29: 27–32.

